# New p65 iso5 isoforms as dexamethasone-binding proteins: novel potential therapeutic targets for inflammatory diseases

**DOI:** 10.3389/fimmu.2026.1748334

**Published:** 2026-03-17

**Authors:** Gaetano Spinelli, Giuseppa Biddeci, Gioacchin Iannolo, Paolo Colomba, Giovanni Duro, Emanuela Maria Marsana, Tommaso Silvano Aronica, Francesco Di Blasi

**Affiliations:** 1 Institute for Biomedical Research and Innovation, National Research Council of Italy (IRIB-CNR), Palermo, Italy; 2 Department of Research, Istituto di Ricovero e Cura a Carattere Scientifico and Istituto Mediterraneo per i Trapianti e Terapie ad alta Specializzazione (IRCCS), Palermo, Italy; 3 Department of Medicine and Surgery, Università degli Studi di Enna “Kore”, Enna, Italy; 4 Complex Operative Unit of Clinical Pathology, Azienda di Rilievo Nazionale ed Alta Specializzazione (ARNAS) Civico Di Cristina e Benfratelli Hospitals, Palermo, Italy

**Keywords:** dexamethasone, glucocorticoid receptor, inflammation, NF-κB, p65 iso5, p65 isoforms

## Abstract

**Introduction:**

The transcription factor NF-κB is a central regulator of immune and inflammatory responses whose activity is tightly controlled by IκB proteins and glucocorticoid receptor (GR)-mediated repression. However, the diversity of NF-κB subunit variants and their contribution to glucocorticoid signaling remain incompletely understood.

**Methods:**

Human peripheral blood mononuclear cells (PBMCs) exposed to pro- and anti-inflammatory stimuli were analyzed to identify splice variants of the NF-κB subunit p65 iso5. mRNA expression was evaluated under different stimuli. Protein interactions with the synthetic glucocorticoid dexamethasone (Dex) and GR were assessed, together with nuclear translocation dynamics. Functional transcriptional activity was examined using NF-κB and IL-2 responsive promoter assays. Expression profiling was also performed in disease contexts, including COVID-19 and liver cirrhosis.

**Results:**

We identified and characterized two previously unrecognized splice variants of p65 iso5, named p65 iso5 Δ6/7 and p65 iso5 Δ10. Their mRNA expression was differentially regulated depending on the stimulus. Both isoforms unexpectedly bound dexamethasone, formed nuclear complexes with Dex-activated GR, and translocated to the nucleus independently of IκBα. Functional assays revealed distinct transcriptional activities on NF-κB and IL-2 responsive promoters, indicating that these isoforms act as noncanonical modulators of glucocorticoid signaling. Expression profiling showed disease-specific regulation, with reciprocal modulation of the two isoforms in COVID-19 and selective upregulation of p65 iso5 Δ6/7 in liver cirrhosis.

**Discussion:**

These findings uncover a previously unrecognized layer of NF-κB/GR crosstalk and identify a new class of dexamethasone-binding proteins outside the nuclear receptor superfamily. Our data highlight their glucocorticoid-interacting properties and provide mechanistic insight into the diversity of glucocorticoid responses, with potential implications for inflammation-related pathologies.

## 1 Introduction

Inflammation is a fundamental physiological response to infection and tissue injury, essential for maintaining cellular homeostasis and defending the host against harmful stimuli. However, dysregulated inflammation can result in chronic inflammatory conditions and contribute to the pathogenesis of autoimmune diseases and cancer (1–3). The Nuclear Factor κB (NF-κB) family comprises ubiquitously expressed transcription factors that orchestrate various aspects of innate and adaptive immunity and serve as central mediators of inflammatory responses (4–6). In mammals, the NF-κB family includes five members: RelA (p65), RelB, c-Rel, and the precursor proteins p105 and p100, which undergo proteolytic processing to form p50 and p52, respectively (7–10). Among these, only p65, c-Rel, and RelB possess C-terminal transactivation domains (TADs), enabling them to initiate transcription. Conversely, p50 and p52 lack TADs but can form transcriptionally active heterodimers with TAD-containing subunits or interact with other transactivating factors. Moreover, homodimers of p50 and p52 can function as transcriptional repressors by competing for κB site binding or by constitutively occupying specific regulatory elements. All NF-κB members share a conserved N-terminal Rel Homology Domain (RHD), which mediates DNA binding, IκB interaction, and nuclear translocation. Structural studies of p50 and p65/p50 dimers have revealed that the amino-terminal region of the RHD facilitates DNA binding, while the C-terminal region supports dimerization and interaction with IκBs (9). Various dimer combinations allow NF-κB to recognize diverse κB consensus sequences with distinct affinities, regulating a broad spectrum of target genes (11). Among these, the p65/p50 heterodimer is the most prevalent and well-studied complex (9). NF-κB activation is mediated through two principal pathways: the canonical and non-canonical pathways (12). The canonical pathway primarily involves p50, RelA, and c-Rel and is associated with cell survival and pro-inflammatory responses (13, 14). In contrast, the non-canonical pathway activates NF-κB2 p52 and RelB, playing roles in lymphoid organogenesis, B cell survival, osteoclastogenesis, and dendritic cell activation (15–18). Aberrant activation of this pathway has been implicated in diseases such as ulcerative colitis, osteoporosis, rheumatoid arthritis, and lymphoid malignancies. Canonical NF-κB signaling is tightly regulated by IκB proteins, which sequester NF-κB dimers in the cytoplasm (9). The IκB family comprises eight members, including IκBα, IκBβ, IκBϵ, IκBζ, IκBNS, Bcl-3, p100, and p105 (5). These proteins contain ankyrin repeat domains that facilitate binding to the RHD of NF-κB subunits, preventing their nuclear translocation (19, 20). In unstimulated cells, p65/p50 heterodimers are retained in the cytoplasm by IκBα (21, 22). Upon stimulation by Toll-like receptors, cytokines, T cell receptors, or cellular stress, the IκB kinase (IKK) complex comprising IKKα, IKKβ, and NEMO is activated (23, 24). Activated IKK phosphorylates IκB, marking it for polyubiquitination and proteasomal degradation. Freed NF-κB dimers then translocate to the nucleus and initiate gene transcription by binding κB sites. NF-κB dysregulation plays a critical role in inflammatory pathologies, autoimmune diseases, atherosclerosis, and various cancers (25, 26) While targeting NF-κB has therapeutic potential, its essential role in immune homeostasis complicates intervention strategies. Nevertheless, exploiting the intricate regulatory mechanisms of NF-κB, such as phosphorylation and interaction with co-factors, may provide more selective therapeutic options (23). The glucocorticoid receptor (GR) and NF-κB represent physiological antagonists in inflammation (27). Glucocorticoids (GCs), including the endogenous hormone cortisol, exert potent anti-inflammatory and immunosuppressive effects by binding to GR, a ubiquitously expressed nuclear receptor (27–31). Upon binding GCs, GR undergoes a conformational change and translocate to the nucleus, where it regulates gene expression via glucocorticoid response elements (GREs) (32, 33). GR also modulates gene expression by interfering with NF-κB through several mechanisms: inducing IκB expression, blocking p65/p50 access to DNA, and competing for coactivators such as CBP and p300. Thus, the crosstalk between NF-κB and GR plays a crucial role in maintaining homeostasis and regulating the inflammatory response (34–37). Despite their efficacy, prolonged GCs use can lead to severe side effects and resistance, necessitating a deeper understanding of their anti-inflammatory mechanisms (35). Alternative splicing contributes significantly to the functional diversity of signaling pathways. The p65 gene has been shown to undergo alternative splicing, generating isoforms such as p65Δ1 (lacking amino acids 222-231), p65Δ2 (lacking amino acids 13-25), and p65Δ3 (lacking amino acids 187-293) (38–40). Recently we identified, a novel isoform termed p65 iso5, containing a previously unrecognized exon (-1) upstream of exon 0, was identified. Remarkably, p65 iso5 binds Dex, a synthetic glucocorticoid, and enhances GR-mediated anti-inflammatory responses. Its expression has been observed in liver tissues of patients with cirrhosis and hepatocellular carcinoma, suggesting disease-specific regulation. Moreover, p65 iso5 mRNA level can be differently expressed in distinct inflammatory related diseases (41, 42). In this study, we report the identification of two additional p65 iso5 isoforms in PBMCs from healthy individuals exposed to inflammatory and anti-inflammatory stimuli. The first, p65 isoform 5 Δ10 (p65 iso5 Δ10), results from an intraexonic splicing event in exon 10, while the second, p65 isoform 5 Δ6/7 (p65 iso5 Δ6/7), arises from skipping exons 6 and 7. Like p65 iso5, these isoforms exhibit Dex-binding capacity and translocate to the nucleus independently of IκBα. These findings further expand the complexity of NF-κB signaling and indicate novel regulatory roles for p65 iso5 Δ6/7 and p65 iso5 Δ10, with potential implications for therapeutic targeting in inflammation-related diseases.

## 2 Materials and methods

### 2.1 Isolation of PBMCs in human samples

Blood samples from 15 healthy subjects were taken for this study. The samples were collected in Vacutainer tubes treated with ethylenediaminetetraacetic acid (EDTA). PBMCs were isolated by centrifugation of whole blood on Ficoll Paque (GE Healthcare), following the manufacturer’s instructions. For the experiments with LPS, the isolated PBMCs were resuspended in DMEM with 10% heat inactivated FBS and then 1.5 x10^6^ cells were plated on 6-well plates and incubated at 37 °C + 5% CO_2_. After 24 h, the PBMCs were treated with 1 μg/ml LPS (O26B6) (Sigma) and incubated for 4 h at 37 °C + 5% CO_2_. This study was conducted in accordance with the Declaration of Helsinki and approved by the Hospital Ethics Committee of the University of Palermo on 14 September 2022 with approval code number 08/2022.

### 2.2 Human liver samples

The study on human liver samples was conducted after obtaining the IRCCS-ISMETT approval (Istituto Mediterraneo per i Trapianti e Terapie ad alta specializzazione - Department of Laboratory Medicine and Advanced Biotechnologies), and patients provided informed consent for research studies. We confirm that the human samples were used in accordance with national guidelines and regulations.

### 2.3 Cell cultures and transfections

HeLa cells (ATCC CCL-2™) were grown at 37 °C under 5% CO_2_ on RPMI 1640 (GIBCO) without antibiotics, supplemented with 5% heat inactivated FBS. COS-1 cells (ATCC CRL-1650™) were grown on DMEM high glucose (Sigma) without antibiotics, supplemented with 10% heat inactivated FBS at 37 °C under 5% CO_2_. 293T (ATCC-CRL-3216 ™) were grown at 37 °C under 5% CO_2_ on DMEM high glucose (Sigma) without antibiotics, supplemented with 10% heat inactivated FBS. All the FRET experiments and fluorescence microscopy were performed using Lipofectamine p300 (Invitrogen) following the manufacturer’s instructions. For luciferase experiments were performed using Polyfect Reagent (Qiagen) following the manufacturer’s instructions, 3x10^5^ HeLa cells/well were plated on 6-well plates and transfected 24 h later with the appropriate concentrations of the specified plasmid. To evaluate the transcriptional activity in heterodimeric complexes, HeLa cells were co-transfected with expression vectors encoding the p65 iso5 isoforms of interest and p50, together with NF-κB or IL-2 luciferase reporter constructs. After 24 h, cells were treated with Dex, and luciferase activity was measured. The relative transcriptional activity of each isoform/p50 heterodimer was calculated by normalizing luciferase signals to Renilla activity, allowing comparison with the canonical p65/p50 dimer. All the experiments were performed in triplicate and replicated at least twice. The quantity of plasmid for each well was kept constant by adding appropriate amounts of the corresponding empty plasmid.

### 2.4 RNA extraction and RT-PCR

Total human RNA was extracted using TRIzol Reagent (Invitrogen). All the experiments were performed in triplicate. The RNA (1 or 2 μg) was reverse transcribed using oligo (dT) primers, RNAsin RNase Inhibitor, M-MLV Reverse Transcriptase and dNTP (Promega). After extraction, the RNA was quantified with a spectrophotometer and electrophoresed on agarose/formaldehyde gel for quality control of the sample. A dilution (1:20) of the RT reaction was used for nested PCR to specifically amplify the entire p65 iso5 mRNA product from PBMCs. In the first PCR reaction, specific primers for exon −1/3′ UTR (forward exon −1 primer 5′- GGAGGGCCTCAGTCGTCCCATC-3′; reverse 3′UTR 5′-AGAATCCGTAAGTGCTTTTGGAGG-3′) were used. The second reaction of nested PCR (a 1:200 dilution of the first PCR reaction) was done with specific primers for exon -1 and reverse exon 10 (5′-AGCTGATCTGACTCAGCAGGGC-3′). Nested PCR products were loaded on agarose gels (1%). PCR products were cloned, and their specificity was confirmed by sequencing. At least three different clones for each PCR product were sequenced.

### 2.5 Real-time quantitative PCR

The oligonucleotides used for qPCR were designed based on the gene sequences taken from the NCBI nucleotide database (GeneBank) and were validated for the absence of secondary structures, self- dimers, as well as primer efficiency and specificity.

For each RT-qPCR reaction, we used 10 μl of ExcelTaq 2X Fast Q-PCR Master Mix (SYBR, ROX) (SMOBIO), 10 pmol/μl of the respective forward and reverse primers, 50 ng of cDNA and RNase- free H_2_O (GIBCO), to a final volume of 20 μl. Human β-actin (ACTB) was used as the housekeeping gene for normalization of gene expression. All cDNA samples were tested in three replicates for housekeeping genes on the same 96-well PCR plate replicated in 40 cycles (95 °C for 20 s, 95 °C for 3 s, 60 °C for 30 s) to reduce possible variations on relative housekeeping genes. Non-template controls and reverse transcription controls were additionally performed. For qPCR, a StepOnePlus Real-Time PCR System (Applied Biosystems, Thermo Fisher) was used with 96-well PCR plates covered with MicroAmp Optical Adhesive Film (Applied Biosystems, Thermo Fisher). All the experiments were performed in triplicate and replicated twice.

### 2.6 Protein sample preparation

The human liver samples have been delivered frozen in cryotubes by 2 ml and stored -80 °C. All procedures were performed at 4 °C on ice. Approximately 25 mg of liver tissue were collected in 500 μl lysis buffer (7 M Urea, 2 M Thiourea, 30 mM CHAPS, 2% Triton X100, 39 mM TRIS pH 8.8, 65 mM DTT, 1 mM Na_3_VO_4_, 1% protease inhibitor cocktail). The tissues have been homogenized 20 times with Dounce (Pestle A), transferred in 2 ml microtubes and centrifuged at 10,000×*g* for 10′ a 15 °C. The supernatant containing the total proteins has been recovered. Protein concentration was determinate by Bradford assay (Bio-Rad).

### 2.7 Western blots

Protein samples were loaded in equal amounts into individual wells of a 10% polyacrylamide-SDS gel, and the proteins were resolved using TRIS-SDS buffer. Proteins were transferred to a nitrocellulose membrane (GE Healthcare) overnight at 4 °C in agitation, and then the nonspecific sites were blocked with 5% BSA in Tris-Buffered Saline pH 7.6 (Tris-HCl and 50 mM 150 mM NaCl) (TBS buffer) containing 0.1% Tween-20 (TBST buffer) with gentle agitation for 30’ at room temperature. The membranes were incubated at room temperature in a fresh solution of 5% BSA-TBST containing the primary antibody: NF-κB p65 (D14E12) Rabbit mAb (1:5000) (Cell Signaling) for 2 h at room temperature. After incubation, membranes were washed twice with TBST (7 min each) and re-incubated with Alexa Fluor 680 goat anti- rabbit IgG 1:10000 (Invitrogen) diluted in 5% BSA-TBST for 1h at room temperature. The membranes were washed three times with TBST (5 min each), and once with TBS. Membranes were scanned and analyzed using the Odyssey infrared imaging system (LI-COR Biosciences, Lincoln, NE, USA) and Odyssey 3.0 imaging software. The band intensities of the experimental target and β-actin was used as the housekeeping protein were quantified by densitometric analysis considering the normalization factor and the level of protein expression is normalized with the mean of the controls. Quantitative analysis of protein bands was performed using ImageJ analysis software.

### 2.8 Flow cytometry-based FRET

Flow cytometry-based FRET measurements were performed using a CytoFLEX SRT Cell Sorter (Beckman) equipped with standard 488 nm and 561 nm lasers. To measure FITC and FRET, cells were excited with the standard 488 nm laser and fluorescence was detected in the green channel with a standard 525/50 nm filter, while the FRET signal was measured with a standard 610/20 nm filter in the channel. For each sample, we analyzed a minimum of 50,000 FITC- and/or mCherry-positive cells according to the gating strategy. All the experiments were performed in triplicate and replicated twice.

### 2.9 Confocal microscopy

For FRET experiments and fluorescence microscopy, 5x10^4^ 293T cells were seeded onto sterile coverslips placed in standard 24-well plates. After 24 hours of incubation, cells were transfected with the appropriate plasmid constructs using Lipofectamine 3000 (Invitrogen), according to the manufacturer’s protocol. Twenty-four hours post-transfection, the culture medium was replaced, and cells were treated with 5 × 10^-7^ M Dex for an additional 24 hours. Following treatment, cells were fixed by incubation with 4% paraformaldehyde (PFA) in 1× PBS for 20 minutes at room temperature in the dark. Subsequently, cells were washed twice with 1× PBS and once with nuclease-free H_2_O (Gibco). Coverslips were mounted using VECTASHIELD^®^ Antifade Mounting Medium (Vector Laboratories) and analyzed using a Leica confocal microscope. Fluorescence imaging was performed using excitation lasers at 405 nm, 488 nm, and 543 nm. DAPI, GFP, and mCherry fluorescence signals were detected using a 63× oil immersion objective. Imaging conditions, including laser intensity, pinhole size, and master/digital gains, were kept constant across all samples to ensure uniformity and reproducibility of fluorescence detection among individual fluorophores and their corresponding fusion proteins. All the experiments were performed in triplicate and replicated twice.

### 2.10 Luciferase assay

HeLa cells were seeded at a density of 1.5x10^5^ on 6-well culture plates containing RPMI and 10% heat inactivated FBS (GIBCO) and incubated at 37 °C + 5% CO2. After 24 h, the cells were transiently co-transfected with 500 ng of different human promotor reporter plasmids and 25 ng of the phRL-TK (Promega) vector encoding Renilla luciferase as internal control. For the experiment, cells were cotransfected with plasmids encoding either human p65, p65 iso5, p65 iso5 Δ6/7, or p65 iso5 Δ10 cDNA (250 ng) under the SV40 promoter. Enzymatic activity was measured 24h after transfection. For all experiments, the total amount of DNA (1.5 μg) for each transfection was kept constant by adding the appropriate quantity of empty plasmid. The enzymatic activity of the two individual reporter enzymes (Firefly and Renilla) was measured in 20 μl of cell lysate with the DualGo Luciferase Reporter Assay System (Promega), according to manufacturer’s instructions, using a GLOMAX (Promega). All the experiments were performed in triplicate and replicated twice.

### 2.11 Statistical analysis

Statistical analyses were performed using GraphPad Prism software (version 7). Comparisons between two groups were conducted using an unpaired two-tailed Student’s t-test. For experiments involving more than two groups, one-way or two-way ANOVA was applied as appropriate, followed by Tukey’s multiple comparisons test. A p-value < 0.05 was considered statistically significant.

## 3 Results

### 3.1 Identification of p65 iso5 Δ6/7 and p65 iso5 Δ10 in human PBMCs

**Figure 1:**
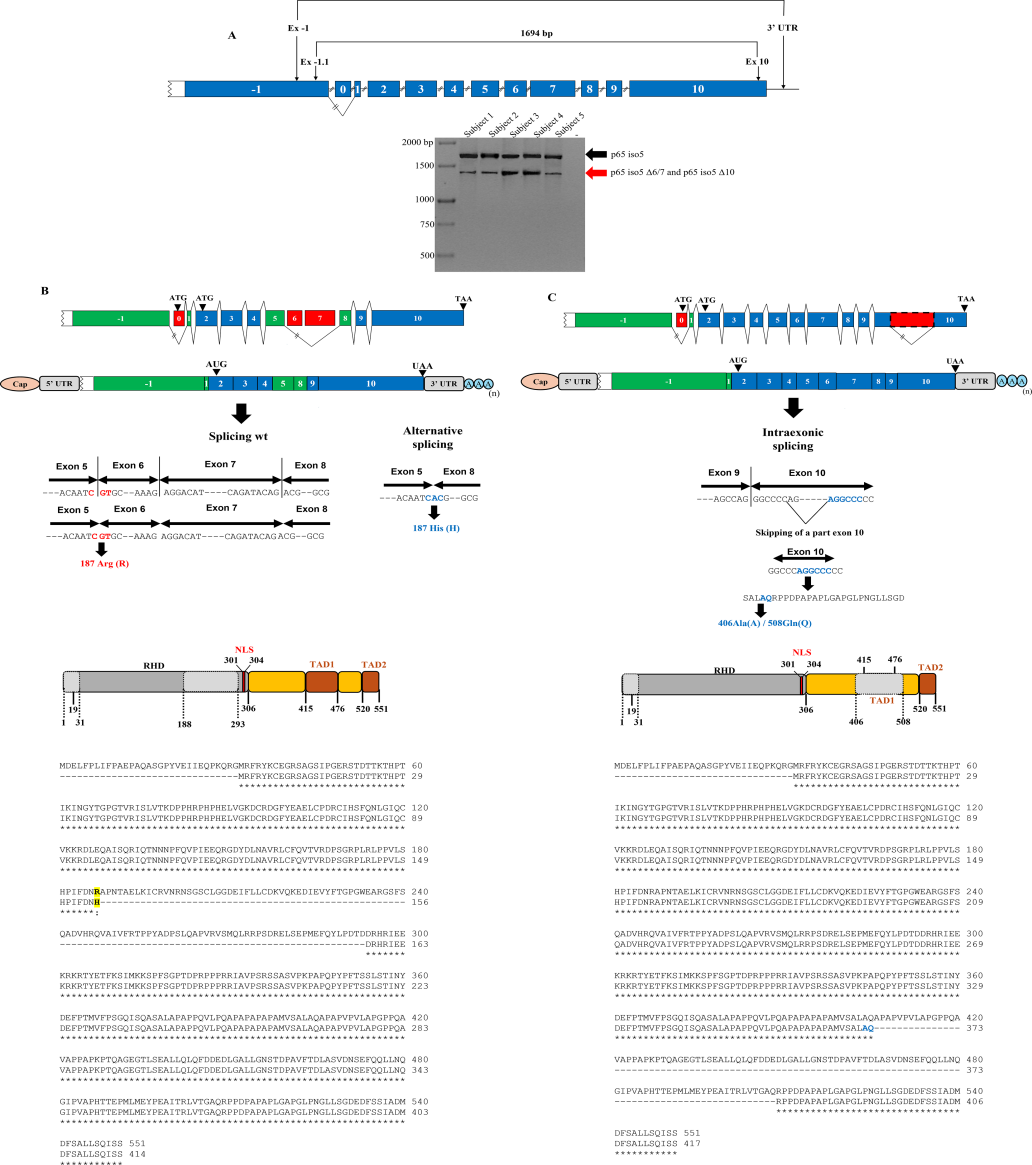
Analysis of alternative splicing events and sequence alignment in PBMCs of healthy subjects. **(A)** First-round PCR reaction was performed with specific oligonucleotides for the exon -1 and 3′ UTR (untranslated region). The second round PCR was performed on first round dilution 1:40 using specific oligonucleotides for exon -1 and exon 10. The - symbol indicates the absence of reverse transcriptase in the reaction (representative of each sample). **(B)** The alternative splicing mechanism in the p65 iso5 Δ6/7, between exons 5 and 8, causes the deletion of exons 6 and 7, with the consequent substitution of the amino acid residue 187. The CGT triplet and the corresponding codon in wt splicing are represented in red and bold, while the CAC triplet and corresponding codon in the p65 iso5 Δ6/7 are in blue and bold. Amino acidic sequences alignment of human p65 and the p65 iso5 Δ6/7. The alignment was performed using the Clustal Omega online tool (EMBL-EBI). The p65 iso5 Δ6/7 is missing 106 amino acids from Ala188 to Asp293. In the alignment, the Arg187His substitution is showed in bold and yellow. The p65 iso5 isoform Δ6/7 ORF is conserved. All amino acids coordinates are referred to p65. **(C)** Intraexonic splicing events in p65 iso5 10. Amino acidic sequences alignment of human p65 and p65 iso5 Δ10. The alignment was performed using the Clustal Omega online tool (EMBL-EBI). All amino acids coordinates are referred to p65.

To evaluate the expression of p65 iso5 in PBMCs from healthy donors, nested PCR was performed using specific primers targeting exon -1 and exon 10 to amplify full-length transcripts. Agarose gel electrophoresis showed the expected p65 iso5 band of 1694 bp corresponding to the previously identified and published p65 iso5 transcript (42) along with an additional ~1400 bp band (**Figure 1A**). As detailed in **Supplementary Information**, **Supplementary Table 1**, the full-length p65 iso5 transcript encodes a predicted protein of 520 amino acids. In contrast, the alternatively spliced isoforms generate shorter transcripts: p65 iso5 Δ6/7 is 1376 bp, predicted to encode a 414 amino acid protein and p65 iso5 Δ10 is 1385 bp, predicted to encode a 417 amino acid protein. Sequence analysis of cloned PCR products confirmed two novel alternatively spliced variants containing exon -1. The first variant, termed p65 iso5 Δ6/7, results from exon skipping of exons 6 and 7, leading to a deletion of 106 amino acids (Ala188 to Asp293) within the RHD. Additionally, canonical splicing at exon 5 (CGT codon encoding Arg187) is replaced by alternative splicing between exons 5 and 8 (CAC codon encoding His187). This substitution does not disrupt the Open Reading Frame (ORF), as shown in the Clustal Omega sequence alignment (**Figure 1B**). GR and NF-κB are inducible transcription factors with opposing roles in the regulation of immune and inflammatory responses (43). Notably, Ser276, a key phosphorylation site for GR-mediated repression of NF-κB, is absent in p65 iso5 Δ6/7, suggesting altered regulatory potential (36, 44). The second variant, p65 iso5 Δ10, arises from an intraexonic splicing event within exon 10, resulting in the loss of 103 amino acids (Ala406 to Gln508) located in the TADs, whereas preserving the ORF (**Figure 1C**).

### 3.2 Expression profiles p65 iso5 Δ6/7 and p65 iso5 Δ10 in PBMCs stimulated with LPS and Dex

**Figure 2:**
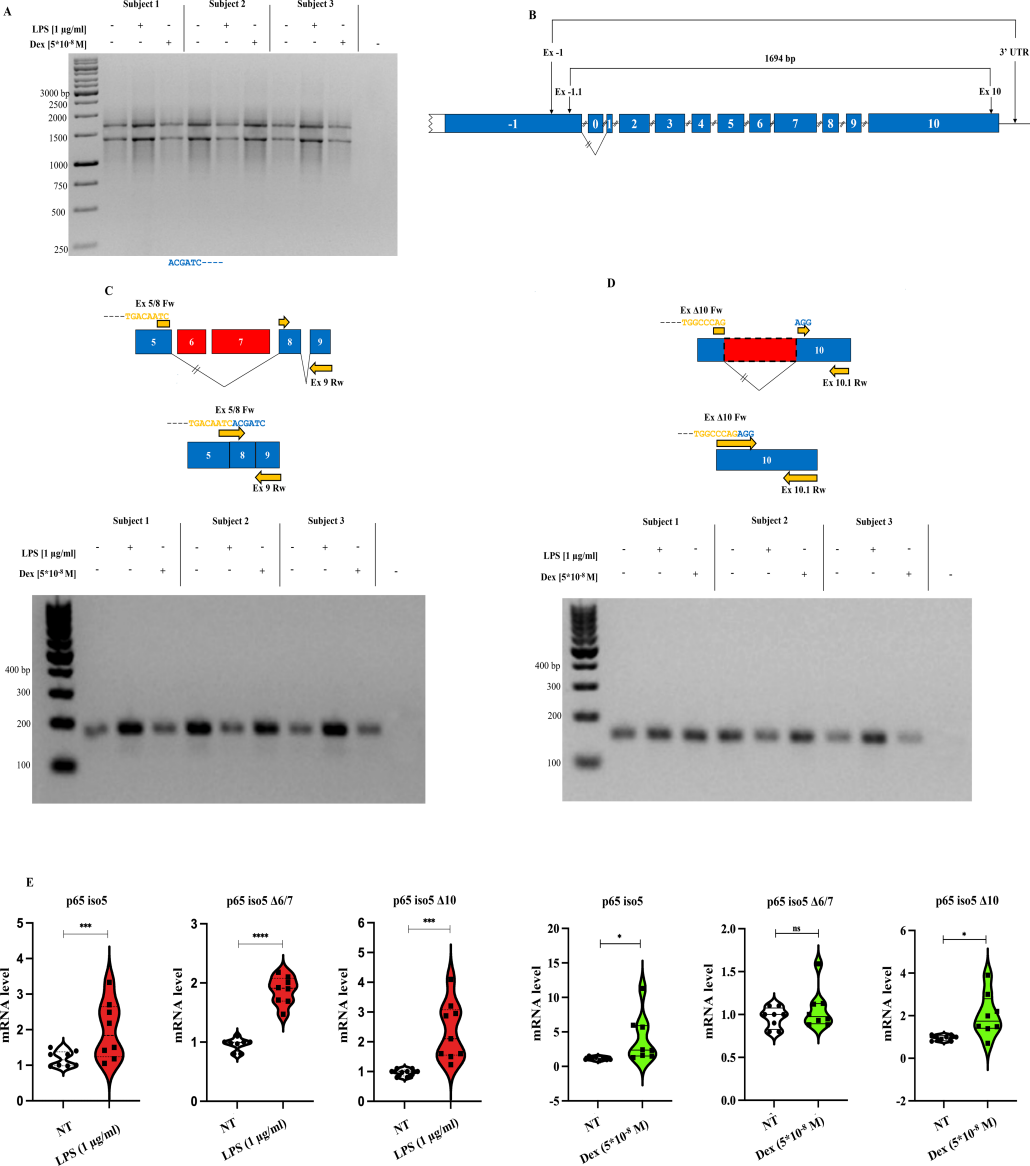
PCR amplification and expression profiling of p65 iso5, p65 iso5 Δ6/7 and p65 iso5 Δ10 in human PBMCs treated with LPS and dex. **(A, B)** First- round PCR reaction was performed with specific oligonucleotides for exon -1 and 3′ UTR. The second-round PCR (on first-round dilution 1:40) was performed with oligonucleotides specific for exons -1 and 10. The symbol - indicates the absence of reverse transcriptase in the reaction (representative of each sample). **(C)** Specific PCR reaction for p65 iso5 Δ6/7 and **(D)** p65 iso5 Δ10. **(E)** The mRNAs expression levels of p65 iso5, p65 iso5 Δ6/7, and p65 iso5 Δ10 in human PBMCs of healthy subjects treated and untreated with LPS [1 µg/ml] and Dex (5*10–^7^ M) for 4h. In **(E)**, data are presented as mean ± SEM ****p < 0.001 ***p < 0.005, * p < 0.05.

Given the central role of NF-κB in inflammatory signaling, we here investigated the expression of p65 iso5, p65 iso5 Δ6/7, and p65 iso5 Δ10 variants in PBMCs following stimulation with lipopolysaccharide (LPS), which triggers a transcriptional response involving multiple inflammation-related genes, and Dex for 4 hours. Nested PCR confirmed the presence of p65 iso5 in all samples and suggested the expression of smaller bands corresponding to alternative splice variants (**Figures 2A, B**). Variant-specific PCR using targeted primers confirmed the expression of both p65 iso5 Δ6/7 and p65 iso5 Δ10 in treated and untreated samples (**Figures 2C, D**; **Table 1**). Quantitative real-time PCR (qPCR) analysis of PBMCs from 15 healthy donors showed that all three isoforms were upregulated in response to LPS. Upon Dex stimulation, p65 iso5 and p65 iso5 Δ10 were significantly induced, whereas p65 iso5 Δ6/7 remained unchanged (**Figure 2E**), indicating isoform-specific regulatory patterns. These results demonstrate that the expression of p65 iso5 Δ6/7 and p65 iso5 Δ10 is dynamically regulated in response to both pro-inflammatory and immunosuppressive stimuli, supporting their potential role in the fine NF-κB signaling. The coordinated upregulation of p65 iso5, p65 iso5 Δ6/7, and p65 iso5 Δ10 following LPS stimulation suggests that these isoforms are integral components of the early inflammatory response. Notably, the selective induction of p65 iso5 and p65 iso5 Δ10 but not p65 iso5 Δ6/7 by Dex indicates differential responsiveness of these variants to GCs signaling, pointing to isoform-specific regulatory mechanisms in the modulation of inflammation.

**Table 1:** Oligonucleotides used in qPCR analysis for p65, p65 iso5, p65 iso5 Δ6/7 and p65 iso5 Δ10.

Target	Forward primer	Reverse primer	Amplified (bp)
p65	ATGGCTCGTCTGTAGTGCACGC	CCGGGAAGATGAGGGGGAAC	98
p65 iso5	AGCCCTGGCTTTGCTCCAGACC	CCGGGAAGATGAGGGGGAAC	115
p65 iso5 Δ6/7	CCCATCTTTGACAATCACGATC	GCTGCGGGAAGGCACAGCAATGC	150
p65 iso5 Δ10	TATCAGCTCTGGCCCAGAGG	TCTGGGGAGGGCAGGCGTCAC	174

### 3.3 p65 iso5 Δ6/7 and p65 iso5 Δ10 drive differential NF-κB signaling and inflammatory gene regulation

**Figure 3:**
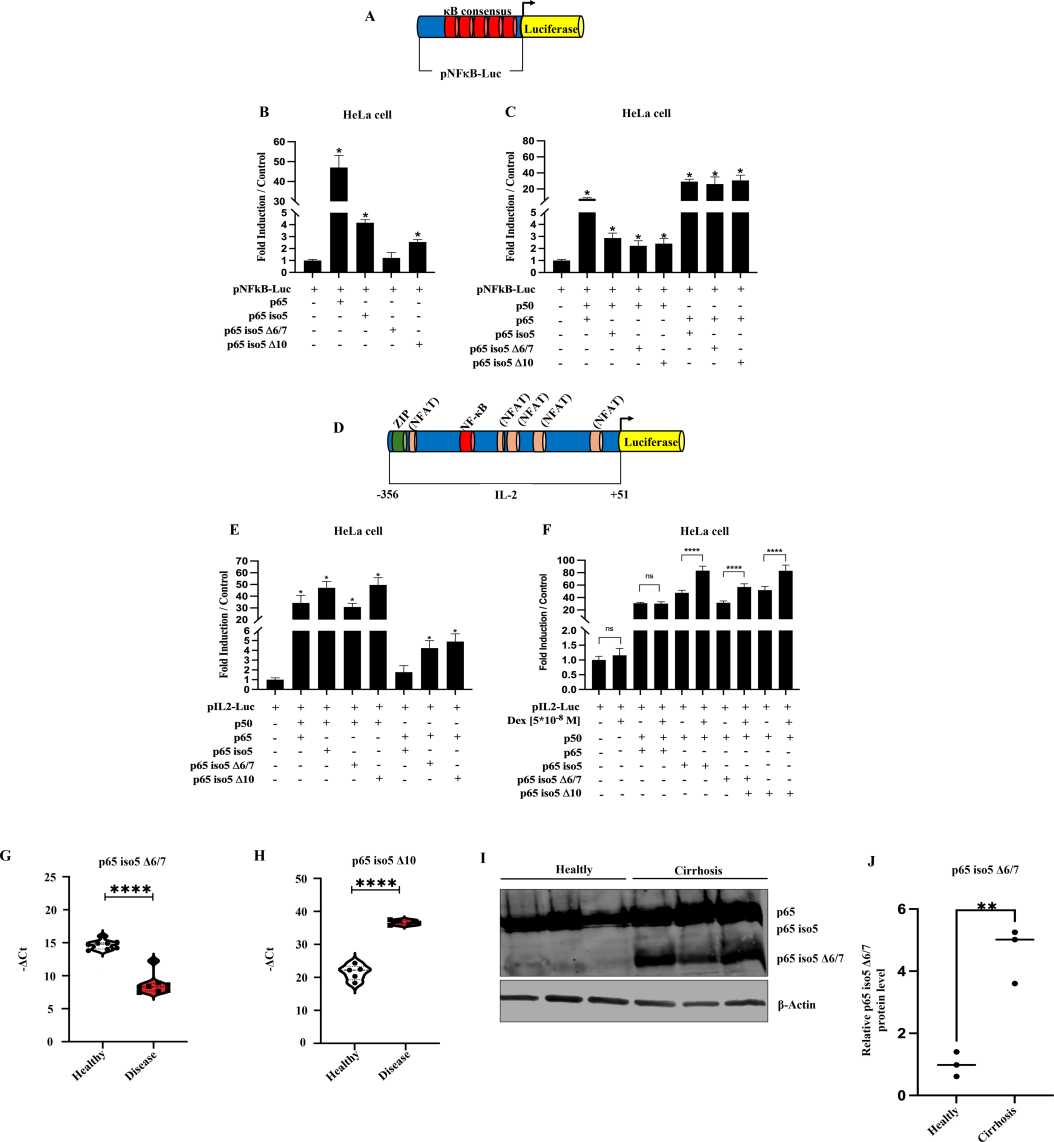
Biochemical activity and disease-associated expression of p65 iso5 Δ6/7 and p65 iso5 Δ10. **(A)** Schematic representation of the artificial NF-κB-Luc promoter, with κB consensus sites shown in red upstream of the luciferase reporter gene. **(B)** Transcriptional activity of p65 iso5, p65 iso5 Δ6/7, and p65 iso5 Δ10 in HeLa cells on the NF-κB-Luc promoter in homodimeric form. **(C)** Transcriptional activity of p65, p50, p65 iso5, p65 iso5 Δ6/7, and p65 iso5 Δ10 in heterodimeric form on the NF-κB-Luc promoter. **(D)** Schematic representation of the natural IL-2 promoter (IL-2-Luc), containing κB consensus sites shown in red upstream of the luciferase reporter gene. **(E)** Transcriptional activity of p65 iso5, p65 iso5 Δ6/7, and p65 iso5 Δ10 in combination with p50 and p65 on the IL-2-Luc promoter. **(F)** Transcriptional activity of p65 iso5, p65 iso5 Δ6/7, and p65 iso5 Δ10 in complex with p50 and p65 on the IL-2-Luc promoter, with or without Dex treatment. **(G)** qPCR analysis of p65 iso5 Δ6/7 and **(H)** p65 iso5 Δ10 expression levels in PBMCs from COVID-19 patients (****p < 0.0001). **(I)** Western blot analysis of liver tissues from healthy controls and cirrhotic patients. **(J)** Densitometric quantification of p65 iso5 Δ6/7 protein levels (**p < 0.01). Data information: In **(B, C, E, F)** data are shown as mean ± SEM. *p < 0.05, ****p < 0.0001, compared to cells transfected with reporter plasmid alone. All experiments were performed in triplicate and replicated at least twice.

To evaluate the biochemical properties and pathophysiological characteristics of p65 iso5 Δ6/7 and p65 iso5 Δ10, we here first investigated their transcriptional activity on NF-κB (**Figure 3A**) and IL-2 (**Figure 3D**) promoters by luciferase reporter assay. On the NF-κB promoter, p65 iso5 Δ6/7 exhibited minimal transcriptional activity, while p65 iso5 Δ10 displayed modest activity both lower than p65 and p65 iso5 (**Figure 3B**). In heterodimeric complexes with p50, all isoforms showed a reduced transcriptional activity compared to the canonical heterodimer p65/p50 (**Figure 3C**). On the IL-2 promoter, p65 iso5/p50 and p65 iso5 Δ10/p50 exhibited significantly higher transcriptional activity than the canonical p65/p50 dimer, in particular when cells were treated with Dex. The heterodimer p65 iso5 Δ6/7/p50 demonstrated activity similar to p65/p50. These results suggest that the p65 iso5 isoforms can modulate cytokine gene expression, especially under GCs influence (**Figures 3E, F**). These data reveal a previously unrecognized functional complexity of these isoforms, indicating that they can modulate the transcriptional activity depending on promoter and stimuli. While p65 iso5 Δ6/7 and p65 iso5 Δ10 show reduced transcriptional activity on the NF-κB promoter, their behavior on the IL-2 promoter reveals a distinct regulatory capacity, suggesting a potential role as specific modulators of cytokine gene expression, especially under GCs influence (**Figures 3E, F**). Moreover, these results showed an additional level of regulation within the NF-κB signaling pathway and highlight the different transcriptional capacity of p65 iso5 Δ6/7 and p65 iso5 Δ10 in gene expression during immune response. Considering these findings, we evaluated the expression profiles of these novel isoforms in clinically relevant inflammatory diseases. In PBMCs isolated from COVID-19 patients, qPCR analyses revealed a significant downregulation of p65 iso5 Δ6/7, coupled with upregulation of p65 iso5 Δ10 compared to healthy subjects (**Figures 3G, H**). This differential expression is consistent with a modulation of NF-κB signaling during acute systemic viral infection and inflammatory responses. Furthermore, western blot analysis of liver tissue from patients affected by cirrhosis showed an upregulation of p65 iso5 Δ6/7 protein (**Figures 3I, J**), indicating a potential association between this isoform and inflammatory-related liver disease. Together, these observations highlight a potential link between isoform expression and inflammatory disease and provide a basis for further investigation to define their functional relevance in pathophysiological contexts.

### 3.4 Dex-binding by p65 iso5 Δ6/7 and p65 iso5 Δ10 variants assessed by FRET-based flow cytometry and confocal microscopy

**Figure 4:**
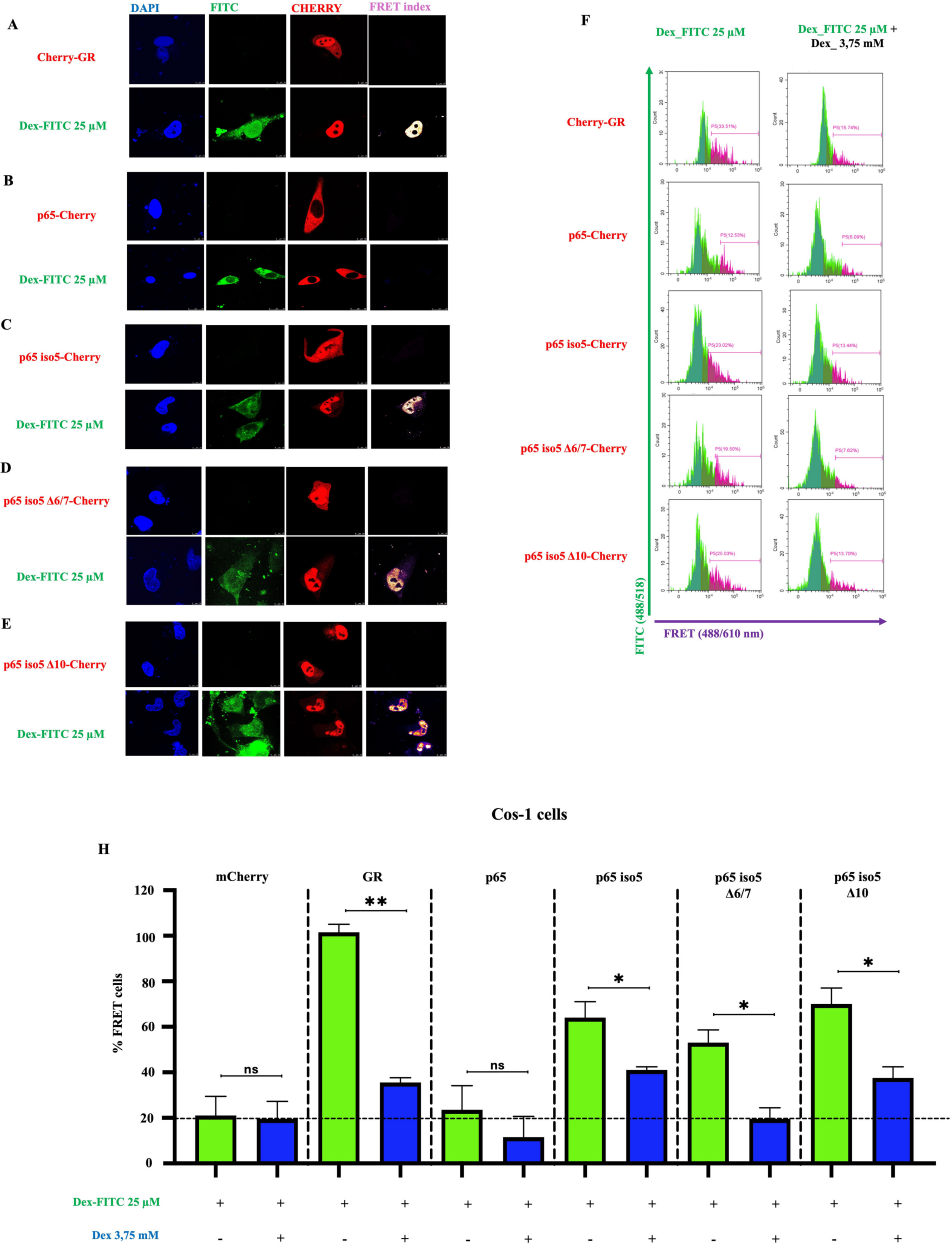
Assessment of p65 iso5 Δ6/7, or p65 iso5 Δ10-dex interaction by FRET and confocal imaging. **(A–E)** Confocal microscopy showed nuclear localization of p65 iso5 Δ6/7-mCherry and p65 iso5 Δ10-mCherry in complex with Dex-FITC. Graphical design for FRET analysis with constructs expressing mCherry-tagged GR, p65, p65 iso5, p65 iso5 Δ6/7, or p65 iso5 Δ10, and treated with Dex-FITC. The Dex-FITC (donor) and mCherry (acceptor). **(F)** FRET-positive cells were identified by plotting FRET *vs*. counts, using cells co-transfected with FITC and mCherry only as negative controls. **(G)** Quantification revealed high FRET signal in mCherry-GR (100%) and p65 iso5 (~60%), with intermediate levels in p65 iso5 Δ6/7 (~50%) and Δ10 (~70%). p65 alone showed minimal FRET signal similar to negative control (~20%). Competitive binding assay with non-fluorescent Dex (3.75 mM) reduced the percentage of FRET-positive cells, confirming specific Dex binding to p65 iso5 p65 iso5 Δ6/7 and p65 iso5 Δ10.

We next examined whether the novel isoforms could bind Dex using FRET via flow cytometry. Dex-FITC has been previously validated as a functional analogue of dexamethasone for GR binding studies, retaining specific GR-binding properties and enabling fluorescence-based detection in competitive assays (45, 46). Constructs expressing mCherry-tagged p65, p65 iso5, p65 iso5 Δ6/7, and p65 iso5 Δ10 isoforms or GR (positive control) were transfected into COS-1 cells and treated with Dex conjugated to fluorescein isothiocyanate (Dex-FITC). As a negative control, cells were co-transfected with vectors expressing FITC and mCherry alone. After 4 hours of treatment, cells were harvested and analyzed by flow cytometry. Live cells were gated based on forward and side scatter (FSC/SSC) parameters, and compensation for spectral overlap between FITC and mCherry was applied. The FRET signal was plotted against FITC intensity, and a gating threshold was established using the FITC/mCherry control to define the FRET-negative population (**Supplementary Information**, **Supplementary Figure 1**). FRET signals were quantified, with the GR-mCherry showing ~100% FRET-positive cells. Wild-type p65 displayed minimal FRET signal (~20%), whereas p65 iso5 reached ~60% positivity. Notably, p65 iso5 Δ6/7 and p65 iso5 Δ10 showed ~50% and ~70% FRET-positive cells respectively. This confirms prior findings that p65 iso5 binds Dex due to an exposed ligand-binding pocket caused by the absence of the first 31 amino acids (42). Competitive inhibition using unlabelled Dex significantly reduced FRET signals, confirming binding specificity for mCherry-GR, p65 iso5, p65 iso5 Δ6/7, and p65 iso5 Δ10 (**Figures 4F, G**). These results demonstrate that structural features, rather than specific amino acid sequences, are critical for Dex-binding activity, even when alternative splicing generates distinct sequences. To further investigate the subcellular localization of Dex/protein complexes, FRET analysis was performed using confocal microscopy. Following Dex-FITC treatment, p65 iso5-mCherry, p65 iso5 Δ6/7-mCherry, and p65 iso5 Δ10-mCherry were predominantly localized in the nucleus (**Figures 4C–E**), similar to GR-mCherry (**Figure 4A**). The nuclear enrichment of the FRET signal suggests that, like GR and p65 iso5, the p65 iso5 Δ6/7 and p65 iso5 Δ10 variants may also participate in GCs-mediated transcriptional regulation. Together, these results demonstrate that p65 iso5 Δ6/7 and p65 iso5 Δ10 can bind Dex and translocate to the nucleus, suggesting these isoforms participate in GR-mediated transcriptional regulation.

### 3.5 Alternative nuclear translocation mechanisms of p65 iso5, p65 iso5 Δ6/7, and p65 iso5 Δ10 in the NF-κB pathway

**Figure 5:**
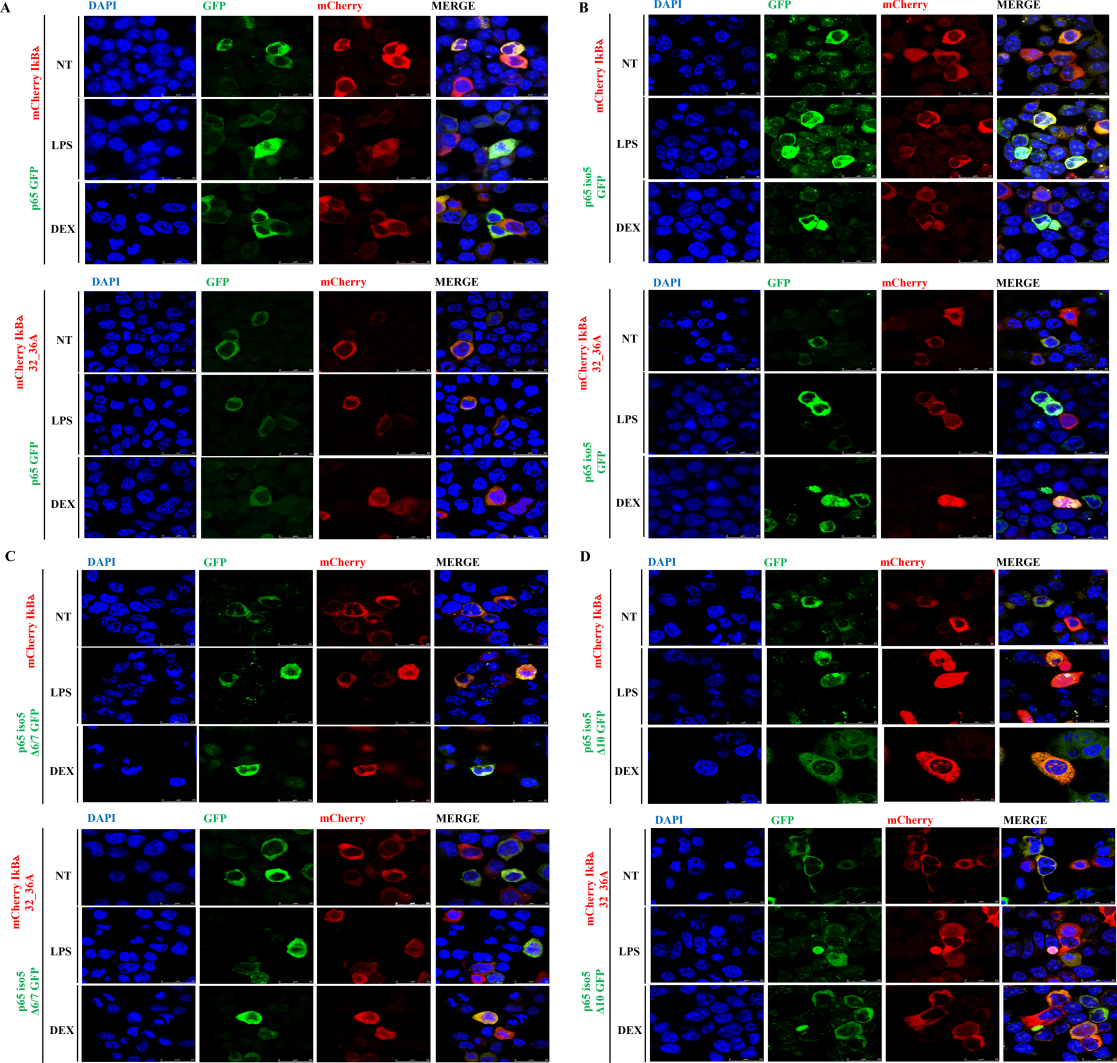
Nuclear localization of p65 isoforms in the presence of wild-type or non-degradable IκBα. 293T cells were co-transfected with constructs encoding GFP-tagged p65 **(A)**, p65 iso5 **(B)**, p65 iso5 Δ6/7 **(C)**, p65 iso5 Δ10 **(D)**, together with either wild-type mCherry-IκBα or the non-phosphorylatable mutant mCherry-IκBα 32-36A. 24 h post-transfection, cells were treated with LPS (1 μg/ml) and Dex (5*10^-7^ M) for 24 h. Confocal microscopy analysis was performed to assess subcellular localization of GF GFP- and mCherry-tagged proteins. Images are representative of at least three independent experiments. Scale bar: 25 μm.

In the canonical NF-κB signaling pathway, the nuclear translocation of p65 is driven by the degradation of IκBα. Under resting conditions, IκBα resides in the cytoplasm, where it binds to and inhibits the p65/p50 dimer. Upon pathway activation, IκBα is phosphorylated at Ser32 and Ser36, leading to its polyubiquitination and subsequent proteasomal degradation. This degradation releases the p65/p50 complex, allowing it to translocate into the nucleus and bind κB response elements to initiate transcription of target genes. Based on confocal fluorescence microscopy experiments showing that p65 iso5 Δ6/7 and p65 iso5 Δ10 can bind Dex and translocate to the nucleus following GCs treatment, we here investigated whether their nuclear translocation depends on IκBα interaction. To this end, 293T cells were co-transfected with constructs encoding p65, p65 iso5, p65 iso5 Δ6/7, or p65 iso5 Δ10 fused at the C-terminus to GFP, along with either wild-type IκBα or a non-phosphorylatable IκBα mutant (32-36A, with Ser32 and Ser36 mutated to alanine), both N-terminally tagged with mCherry. Following transfection, cells were treated with LPS and Dex and analyzed by confocal microscopy using appropriate filters for GFP and mCherry. As expected, p65-GFP localized to the nucleus upon LPS stimulation in the presence of wild-type mCherry-IκBα, whereas Dex treatment alone did not induce nuclear translocation. Moreover, co-expression of p65-GFP with mCherry-IκBα 32-36A prevented nuclear localization even after LPS and Dex treatment, consistent with the mutant’s inability to undergo phosphorylation and degradation (**Figures 5A, B**). Interestingly, co-expression of p65 iso5-GFP, p65 iso5 Δ6/7-GFP, or p65 iso5 Δ10-GFP with either mCherry-IκBα or mCherry-IκBα 32-36A resulted in a marked nuclear localization of these isoforms following combined LPS and Dex treatment (**Figures 5C, B**). These findings indicate that these isoforms are capable of translocating to the nucleus even in the presence of a non-degradable IκBα variant. Taken together, these results indicate that p65 iso5, p65 iso5 Δ6/7, and p65 iso5 Δ10 may utilize non-canonical regulatory mechanisms distinct from classical NF-κB signaling. In particular, their ability to respond to both pro- and anti-inflammatory stimuli and to localize to the nucleus under conditions that block canonical IκBα-mediated translocation suggests they may engage alternative signaling pathways or interact with different molecular partners. This highlights an additional layer of complexity in the regulation of the inflammatory response.

### 3.6 Live-cell FRET analysis of GR interaction with either p65 iso5, p65 iso5 Δ6/7 and p65 iso5 Δ10 in response to Dex

**Figure 6:**
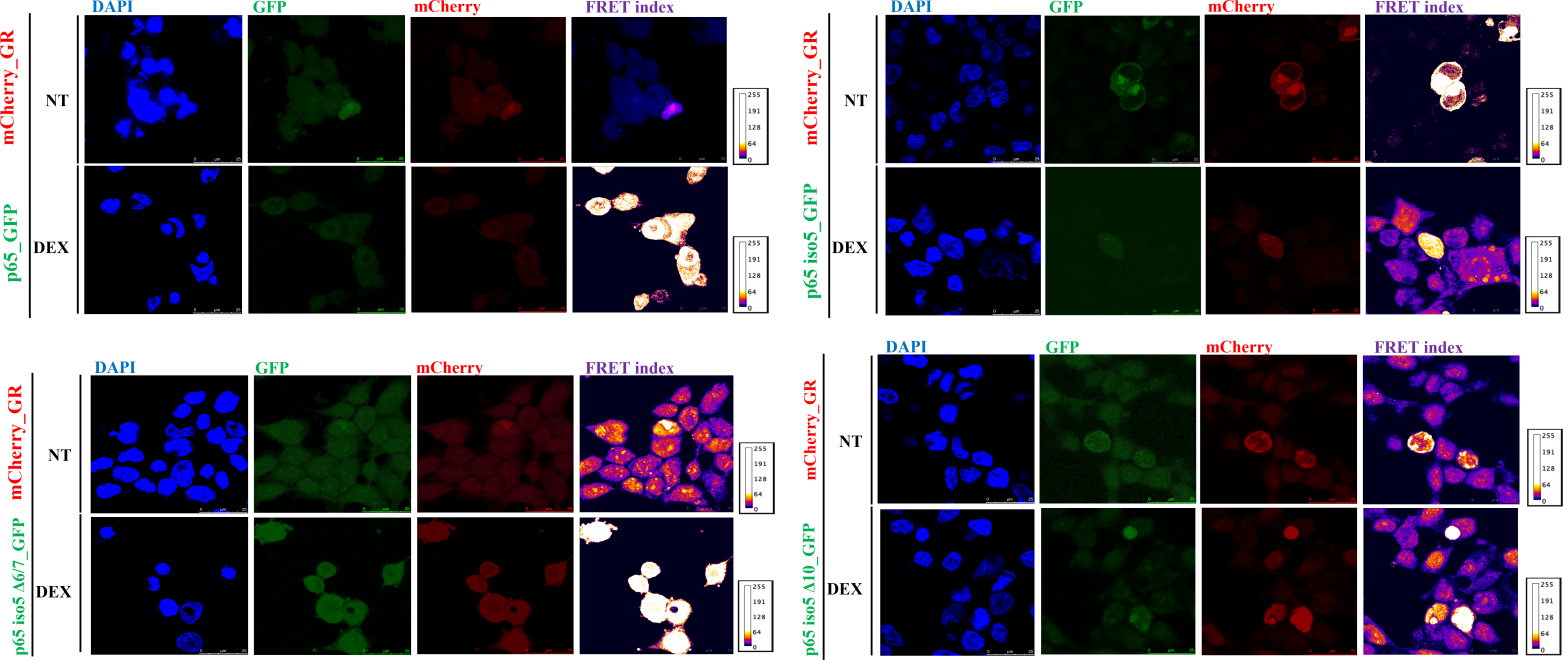
FRET-based analysis of GR interaction with p65 iso5 isoform upon dex stimulation in living cells. 293T cells were transfected with C-terminal GFP-tagged p65 iso5, p65 iso5 Δ6/7, or p65 iso5 Δ10 constructs, together with N-terminal mCherry-tagged GR. FRET efficiency was assessed by confocal microscopy using a 488 nm laser for GFP excitation and a 610 nm detector for mCherry emission, capturing energy transfer events. Representative confocal images of 293T cells co-expressing the indicated constructs under basal conditions and after treatment with 5×10^-7^ Dex for 24 h. Scale bar, 25 µm.

To investigate the potential interaction between the GR and the p65 iso5, p65 iso5 Δ6/7, and p65 iso5 Δ10 isoforms in response to Dex, live-cell FRET-based protein-protein interaction analyzed were performed using confocal microscopy. As a baseline control, 293T cells were transfected with GFP alone to establish background donor fluorescence, which was subsequently used to calculate the FRET index under experimental conditions. Following background acquisition, cells were co-transfected with constructs encoding C-terminal GFP-tagged p65 iso5, p65 iso5 Δ6/7, or p65 iso5 Δ10, along with an N-terminal mCherry-tagged GR. After Dex treatment, cells co-expressing GFP and mCherry were imaged using the appropriate fluorescence channels. FRET efficiency was quantified using a dedicated ImageJ plugin, based on excitation at 488 nm (GFP) and emission detection at 610 nm (mCherry), thereby capturing energy transfer events indicative of protein–protein interactions (**Supplementary Information**, **Supplementary Figure 2**). No significant FRET signal was detected in cells expressing wild-type p65-GFP and mCherry-GR, even after Dex treatment. In contrast, Dex stimulation induced a robust FRET signal in cells co-expressing GR and any of the p65 iso5 variants, suggesting a direct interaction between these isoforms and GR, both in the cytoplasm and nucleus (**Figure 6**). These findings are consistent with prior reports indicating that activated GR can physically interact with p65 to inhibit NF-κB signaling by preventing its dimerization with p50. Interestingly, in the absence of Dex, cells expressing p65 iso5-GFP, p65 iso5 Δ6/7-GFP, or p65 iso5 Δ10-GFP together with mCherry-GR exhibited a basal cytoplasmic FRET signal, indicating a constitutive interaction between these isoforms and GR. Upon Dex stimulation, both FRET efficiency and the nuclear localization of the GR/isoform complexes increased significantly (**Figure 6**). Of note, the p65 iso5 Δ6/7 variant lacks the Ser276 residue (numbering based on the canonical p65 sequence), which is considered critical for GR binding. Nevertheless, this isoform retained the ability to interact with GR and translocate to the nucleus upon Dex treatment. As previously suggested by Lambourne et al., structural changes in protein folding may expose alternative interaction surfaces. Accordingly, the unexpected interaction between the p65 iso5 Δ6/7 isoform and GR, despite the absence of Ser276, may be explained by altered folding that reveals novel binding interfaces (47). Collectively, these results demonstrate that p65 iso5, p65 iso5 Δ6/7, and p65 iso5 Δ10 can interact with Dex-activated GR and form nuclear complexes. These interactions indicate a potential role for these isoforms in modulating GR-mediated anti-inflammatory responses and highlight them as possible therapeutic targets in combination with GCs for the treatment of inflammatory diseases.

## 4 Discussion

Our results reveal that the NF-κB pathway harbors previously unrecognized isoforms of the p65 iso5 transcript with the unexpected ability to bind the glucocorticoid Dex. This finding reshapes the current understanding of NF-κB signaling by uncovering a direct molecular interface between pro-inflammatory and glucocorticoid-mediated anti-inflammatory pathways. Given the central role of NF-κB dysregulation in diverse immune-mediated diseases, including autoimmune disorders, cancer, chronic inflammatory conditions, viral infections (13, 48), glucocorticoid-responsive p65 iso 5 splice variants may contribute to disease-specific patterns of immune activation and variable steroid responsiveness. NF-κB and GR have long been viewed as functional antagonists that finely balance pro- and anti-inflammatory gene expression (49). The identification of p65 iso5 Δ6/7 and p65 iso5 Δ10 as novel NF-κB splice variants capable of binding Dex and translocating to the nucleus independently of IκBα represents a paradigm shift in our understanding of inflammatory regulation, showing that certain NF-κB components can directly participate in glucocorticoid signaling. These findings expand the conceptual framework of inflammatory regulation and provide mechanistic insight into how transcriptional crosstalk between NF-κB and GR can be achieved through alternative splicing. The inflammatory relevance of these isoforms is underscored by their stimulus-specific expression and transcriptional behavior. Both isoforms are upregulated in PBMCs following LPS stimulation, indicating their involvement in early inflammatory responses. However, their differential regulation by Dex selective induction of p65 iso5 and p65 iso5 Δ10, but not p65 iso5 Δ6/7 suggests isoform-specific responsiveness to glucocorticoid signaling. This divergence may reflect distinct roles in modulating inflammation under immunosuppressive conditions and points to a nuanced regulatory mechanism beyond classical GR-mediated repression. Previous studies have shown that mutations at specific amino acid residues (50, 51), or deletions (52) within the RHD of p65 can impair its interaction with regulatory partners. Both p65 iso5 Δ6/7 and p65 iso5 Δ10 originate from transcripts containing the noncanonical exon -1 previously described in p65 iso5, but they feature additional deletions that reshape critical structural domains within the RHD and TADs. Despite these deletions, both isoforms preserve an open reading frame and acquire the ability to bind Dex with high specificity, as demonstrated by FRET-based assays and competitive inhibition with unlabeled Dex. These results indicate that structural remodeling rather than canonical ligand-binding motifs governs Dex recognition. The emergence of ligand-binding pockets in NF-κB isoforms suggests a new mode of steroid-hormone responsiveness independent of classical nuclear receptors. A remarkable property of these isoforms is their IκBα-independent nuclear translocation. Canonical p65 requires IκBα degradation for nuclear import, but p65 iso5 Δ6/7 and p65 iso5 Δ10 retained nuclear localization even in the presence of a non-degradable IκBα mutant. This implies the existence of alternative import pathways or unique protein-protein interactions that bypass the classical IKK-IκB-NF-κB axis. Such an ability may enable rapid transcriptional responses to glucocorticoids or inflammatory cues, offering a plausible explanation for tissue-specific differences in steroid sensitivity. Functionally, these isoforms exert distinct transcriptional effects. On synthetic NF-κB promoters the two isoforms display attenuated transcriptional activity, whereas on the IL-2 promoter they exhibit a marked enhancement, particularly in the presence of Dex. This promoter- and context-dependent behavior indicates that p65 iso5 Δ6/7 and p65 iso5 Δ10 may reprogram the inflammatory transcriptional landscape by selectively modulating cytokine expression in a glucocorticoid-responsive manner. Their enhanced activity on the IL-2 promoter is especially relevant from an immunological perspective, as it suggests that these variants could influence T-cell activation thresholds and effector differentiation under steroid exposure (53). The p65 iso5 Δ10 isoform maintains the residues required for potential interaction with p50 and is therefore capable of forming heterodimers (54). In contrast, p65 iso5 Δ6/7 lacks specific structural domains necessary for full interaction with p50. Consistently, in our transcriptional assays on the IL-2 promoter, the transcriptional activity of the p65 iso5 Δ6/7-p50 complex was lower than that of the p65 iso5 Δ10-p50 complex, even in the presence of Dex. Although we did not directly assess whether the affinity of these isoforms for p50 is modulated under basal or LPS/Dex-stimulated conditions, this represents an important question for future investigation. Elucidating the stimulus-dependent dynamics of these heterodimeric complexes may further clarify how alternative splicing influences NF-κB dimer composition and transcriptional specificity in inflammatory and glucocorticoid-responsive contexts. This mechanism could suggest a probable molecular basis for glucocorticoid responsiveness and highlights how alternative NF-κB isoforms may fine-tune adaptive immune responses during inflammation. Moreover, the opposite regulation of these isoforms in COVID-19, characterized by decreased p65 iso5 Δ6/7 and increased p65 iso5 Δ10, along with the elevated p65 iso5 Δ6/7 expression observed in liver cirrhosis, indicates that they may have distinct, disease-specific functions. These findings suggest that NF-κB alternative splicing contributes to fine-tuning inflammatory responses across distinct pathological contexts. The differential expression observed in PBMCs from COVID-19 patients and in cirrhotic liver tissue further supports the notion that these isoforms may contribute to disease-specific modulation of inflammatory pathways. While the present study provides direct experimental evidence in three clinically relevant contexts, broader transcriptomic analyses across large patient cohorts could substantially expand our understanding of the distribution and regulation of these isoforms in additional inflammatory or immune-mediated conditions. Such large-scale RNA-seq-based investigations represent an important future direction arising from the hypotheses generated by our results and may help clarify the translational potential of these noncanonical Dex-binding NF-κB variants. At a mechanistic level, our results indicate that these p65 iso5 isoforms can physically interact with activated GR, forming cytoplasmic complexes that translocate into the nucleus upon Dex stimulation. Such interactions may expand the repertoire of GR-regulated genes by recruiting NF-κB isoforms to novel genomic loci or by modulating coactivator usage. This partnership provides a potential explanation for one of the longstanding paradoxes in glucocorticoid biology, namely how glucocorticoids can simultaneously repress pro-inflammatory NF-κB activity while promoting selective anti-inflammatory gene expression (55). The ability of p65 iso5 Δ6/7 and p65 iso5 Δ10 to act as Dex-binding co-regulators suggests a conceptual framework for how these isoforms could modulate transcriptional crosstalk between NF-κB and GR, providing a basis for future exploration of their potential as modulators of glucocorticoid responses. From a translational perspective, the identification of Dex-binding NF-κB isoforms opens new opportunities for therapeutic intervention in chronic inflammation and glucocorticoid-resistant diseases. However, these therapeutic implications should currently be considered hypothesis-generating. Further mechanistic validation and *in vivo* studies will be required to determine whether selective targeting of these isoforms is feasible and clinically relevant. At this stage, p65 iso5 Δ6/7 and p65 iso5 Δ10 could be considered potential therapeutic targets whose selective modulation may provide new opportunities for isoform-specific regulation of NF-κB signaling and guide the development of future pharmacological strategies. The discovery of these Dex-binding p65 iso5 isoforms highlights a previously unappreciated layer of complexity within the NF-κB network and the GR pathway. Unlike conventional glucocorticoid therapy, which relies exclusively on GR activation, these isoforms retain NF-κB structural motifs while acquiring Dex responsiveness, suggesting a conceptual framework for selective modulation of NF-κB signaling. Collectively, our findings indicate that alternative splicing generates steroid-responsive NF-κB variants capable of bridging innate immune signaling and hormonal control, providing mechanistic insight into the diversity of glucocorticoid responses. In conclusion, our study identifies a new class of Dex-binding NF-κB isoforms that operate at the intersection of inflammatory and hormonal signaling. By demonstrating that p65 iso5 Δ6/7 and p65 iso5 Δ10 directly interact with the GR and modulate transcription in an IκBα-independent manner, we provide a molecular basis for selective glucocorticoid responsiveness. These findings expand the current paradigm of steroid action, highlight alternative splicing as a key regulatory mechanism in inflammation, and open avenues for the development of isoform-specific therapeutics aimed at controlling chronic inflammatory and immune-mediated diseases.

## Data Availability

The datasets presented in this study can be found in online repositories. The names of the repository/repositories and accession number(s) can be found in the article/**Supplementary Material**.

## Supplementary material

Supplementary Figure 1 FRET analysis between FITC and mCherry fluorophores in Cos-1 cells. **(A)** Spectral overlap analysis using the FPbase FRET Calculator demonstrates a significant overlap between the emission spectrum of FITC and the excitation spectrum of mCherry, suggesting suitability for FRET pairing. Key parameters such as Förster distance (R_0_ = 5.9 nm) and spectral overlap integral (J(λ) = 2.46 × 10^15^ M^−1^ cm^−1^ nm^4^) are shown. **(B)** Fluorescence microscopy images of Cos-1 cells treated with 25 µM Dex-FITC or expressing mCherry-tagged constructs. DAPI stains nuclei (blue), Dex-FITC signals (green), mCherry (red), and FRET signals (magenta) are shown. No significant FRET was detected, suggesting insufficient proximity or interaction between the two fluorophores in this context. **(C)** Flow cytometry analysis of FRET efficiency. FITC fluorescence (488/518 nm) and FRET signal (488/610 nm) were measured in Cos-1 cells co-expressing Dex-FITC (25 µM) with or without unlabeled Dex (0.125 M). No significant FRET was detected, suggesting insufficient proximity or interaction between the two fluorophores in this context.

Supplementary Figure 2 Evaluation of EGFP-mCherry FRET in 293T cells. **(A)** Spectral analysis using the FPbase FRET Calculator demonstrates overlap between the emission spectrum of EGFP (donor) and the excitation spectrum of mCherry (acceptor), indicating potential for FRET. Calculated Förster radius (R_0_) is 5.29 nm, with a spectral overlap integral J(λ) = 1.94 × 10^15^ M^−1^ cm^−1^ nm^4^. **(B)** Fluorescence microscopy of 293T cells transfected with plasmids encoding pEGFP, pmCherry, or untransfected control. Nuclei are stained with DAPI (blue). GFP (green), mCherry (red), and FRET index (magenta) channels are shown. Cells expressing both GFP and mCherry were assessed for FRET signal. No significant FRET was detected, suggesting insufficient proximity or interaction between the two fluorophores in this context.

Supplementary Table 1 Nucleotide and predicted amino acid lengths of p65 iso5 isoforms. The table summarizes the nucleotide length in base pairs and the predicted protein length in amino acids of the full-length p65 iso5 transcript and its alternatively spliced variants p65 iso5 Δ6/7 and p65 iso5 Δ10. The p65 iso5, 1694 bp, encodes a 520 amino acid protein, whereas the isoforms p65 iso5 Δ6/7 and p65 iso5 Δ10 generate shorter transcripts of 1376 bp and 1385 bp, predicted to encode proteins of 414 and 417 amino acids, respectively.
