# Supplementary figures and images for: New p65 iso5 isoforms as dexamethasone-binding proteins: novel potential therapeutic targets for inflammatory diseases
Source: Front Immunol. 2026 Mar 17;17:1748334. doi: 10.3389/fimmu.2026.1748334 (PMC13036746; doi:10.3389/fimmu.2026.1748334)

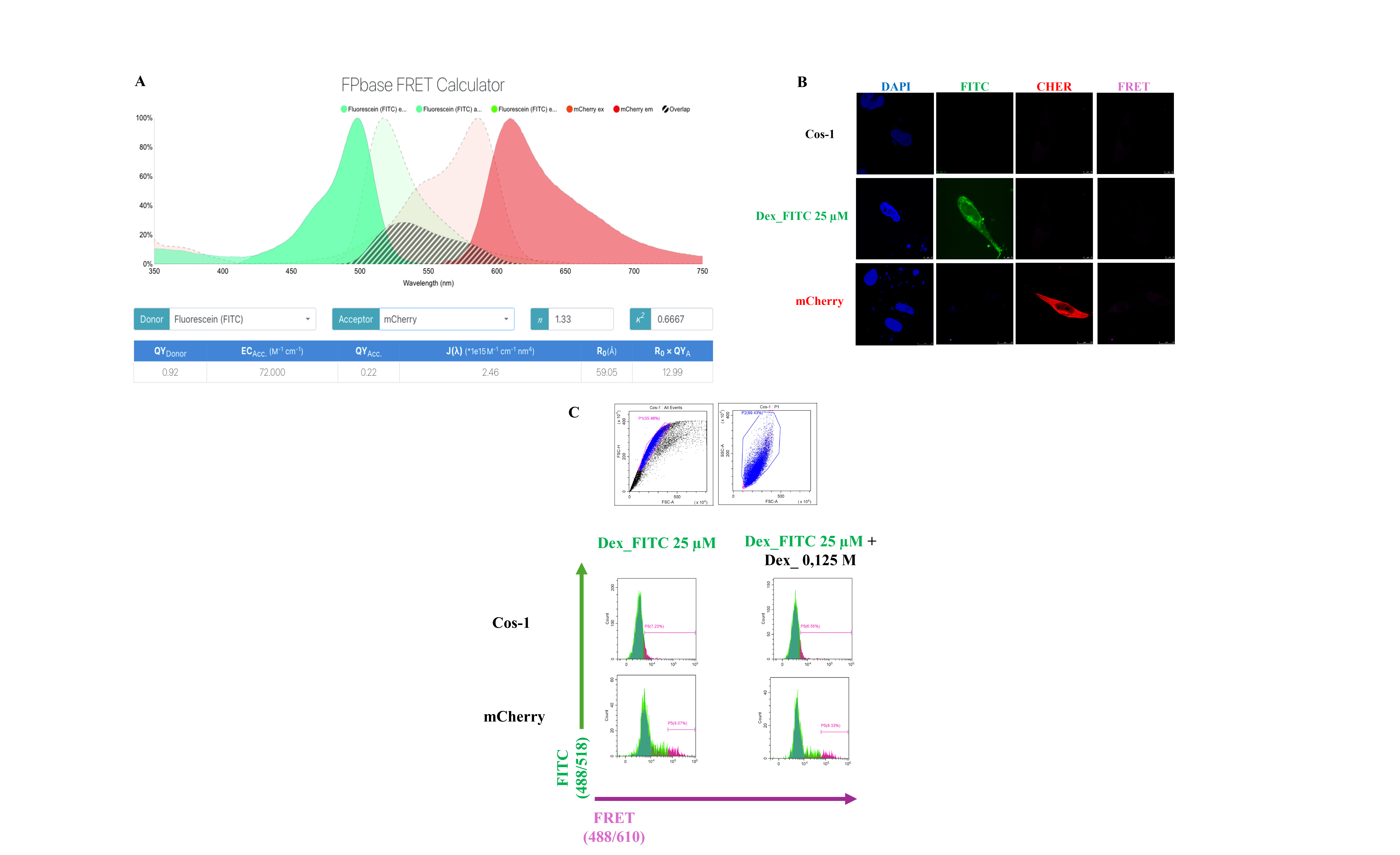

Supplement: Supplementary Figure 1 — FRET analysis between FITC and mCherry fluorophores in Cos-1 cells. (A) Spectral overlap analysis using the FPbase FRET Calculator demonstrates a significant overlap between the emission spectrum of FITC and the excitation spectrum of mCherry, suggesting suitability for FRET pairing. Key parameters such as Förster distance (R0 = 5.9 nm) and spectral overlap integral (J(λ) = 2.46 × 10^15^ M−1 cm−1 nm4) are shown. (B) Fluorescence microscopy images of Cos-1 cells treated with 25 µM Dex-FITC or expressing mCherry-tagged constructs. DAPI stains nuclei (blue), Dex-FITC signals (green), mCherry (red), and FRET signals (magenta) are shown. No significant FRET was detected, suggesting insufficient proximity or interaction between the two fluorophores in this context. (C) Flow cytometry analysis of FRET efficiency. FITC fluorescence (488/518 nm) and FRET signal (488/610 nm) were measured in Cos-1 cells co-expressing Dex-FITC (25 µM) with or without unlabeled Dex (0.125 M). No significant FRET was detected, suggesting insufficient proximity or interaction between the two fluorophores in this context. [file Image1.tif]

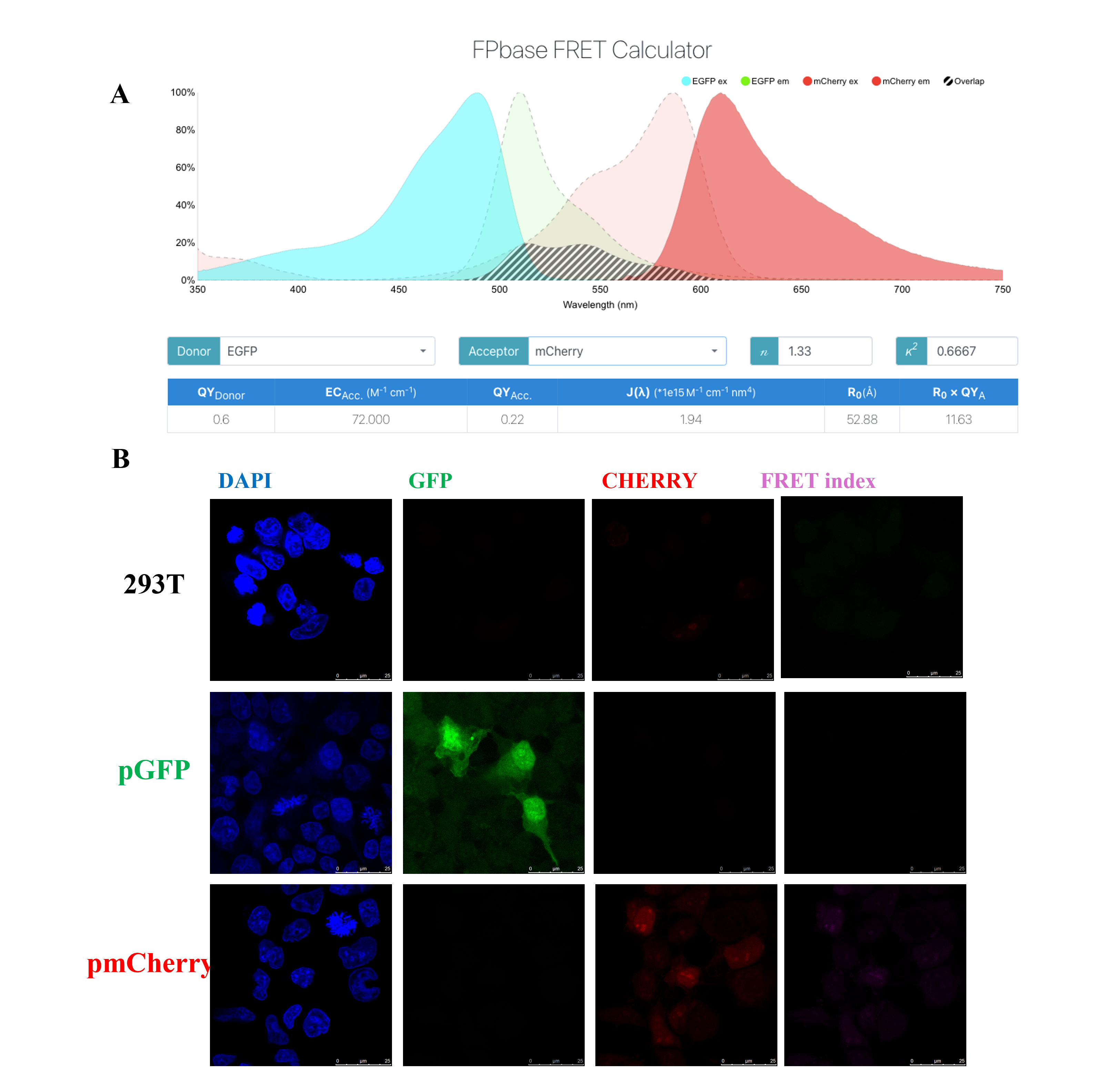

Supplement: Supplementary Figure 2 — Evaluation of EGFP-mCherry FRET in 293T cells. (A) Spectral analysis using the FPbase FRET Calculator demonstrates overlap between the emission spectrum of EGFP (donor) and the excitation spectrum of mCherry (acceptor), indicating potential for FRET. Calculated Förster radius (R0) is 5.29 nm, with a spectral overlap integral J(λ) = 1.94 × 10^15^ M−1 cm−1 nm4. (B) Fluorescence microscopy of 293T cells transfected with plasmids encoding pEGFP, pmCherry, or untransfected control. Nuclei are stained with DAPI (blue). GFP (green), mCherry (red), and FRET index (magenta) channels are shown. Cells expressing both GFP and mCherry were assessed for FRET signal. No significant FRET was detected, suggesting insufficient proximity or interaction between the two fluorophores in this context. [file Image2.tif]

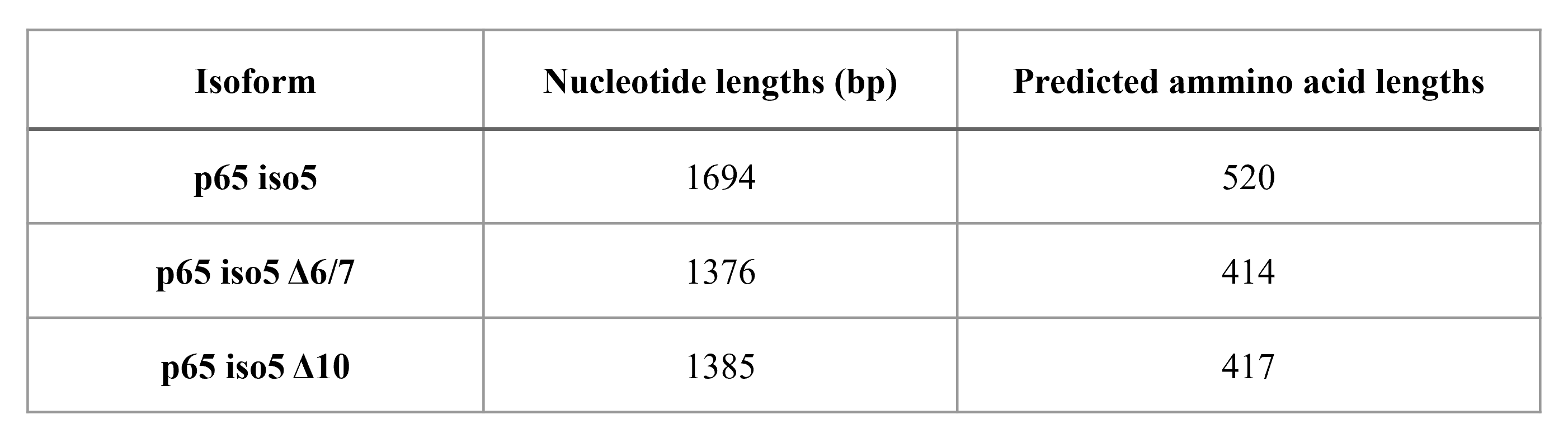

Supplement: Supplementary Table 1 — Nucleotide and predicted amino acid lengths of p65 iso5 isoforms. The table summarizes the nucleotide length in base pairs and the predicted protein length in amino acids of the full-length p65 iso5 transcript and its alternatively spliced variants p65 iso5 Δ6/7 and p65 iso5 Δ10. The p65 iso5, 1694 bp, encodes a 520 amino acid protein, whereas the isoforms p65 iso5 Δ6/7 and p65 iso5 Δ10 generate shorter transcripts of 1376 bp and 1385 bp, predicted to encode proteins of 414 and 417 amino acids, respectively. [file Table1.tif]
